# Psychometric analysis of the emotional availability scales for video-recorded interactions between parents and their preschool-aged children

**DOI:** 10.3389/frcha.2025.1528196

**Published:** 2025-04-09

**Authors:** Jörg Michael Müller, Christina Elvert

**Affiliations:** Department of Child and Adolescent Psychiatry, University Hospital Münster, Münster, Germany

**Keywords:** emotional availability scales, parent-child interaction, preschool age, psychometric analysis, assessment

## Abstract

In the context of parent-child interaction, the Emotional Availability Scales have been developed to capture a dyad's emotional connection in an observational setting by four parental and two child-related scales. This study aims to test the psychometric foundation of the EAS, including basic descriptive preconditions on the item level and structural validity on the scale level, for a preschool-aged sample; as such, it complements analyses by Aran for a sample of infants. The sample of parents and their preschool-aged children is a mixed clinically referred and non-clinical sample from a midsize city in Germany. Interactions were observed in a free-play setting and rated with the EAS by two blind and certificated raters. Several model tests indicate violations for the structural model as well as all six measurement models. An additional *post hoc* exploratory factor analysis with parallel analysis suggests a non-interpretable two-factor structure. Psychometric analyses did not validate the EAS's postulated structure and measurement model. A *post hoc* literature review showed that ceiling effects on the item and scale levels are not unique to our study. However, traditionally important concepts or terms of parent-child relationships covered by the EAS are not rejected by our study and can be assessed with alternative measures, but these also need psychometric evaluation in the future.

## Introduction

The Emotional Availability Scales (1) represent a widely known instrument (2) that promises to cover central aspects of a parent-child relationship by four parental scales (sensitivity, structuring, non-intrusiveness, and non-hostility) and two child scales [responsiveness and involvement (1)]. The EAS origin is based on terms coined by Ainsworth (3), such as maternal sensitivity/insensitivity, interference/cooperation, acceptance/rejection (4), and accessibility/inaccessibility. Assessing several scales is reasonable if each scale is informative in the sense of providing additional information not assessed by the remaining scales. This implies that the scales have to be sufficiently independent. In contrast to this assumption, studies have consistently reported relatively high to extremely high intercorrelations for the EAS (5). The sizes of theses correlations introduce doubt about whether the six scales are empirically discriminable. Because a psychometric analysis including exploratory or confirmatory factor analysis has not been reported yet, profound interpretation of the six-scale scores is not supported (6). Aran et al. (7) examined the postulated factor structure introduced by Biringen (8) and concluded that one latent factor underlies all six scales, which is in line with other findings (9).

In general, observational instruments are—in contrast to questionnaire-based assessments (10)—seldom examined with respect to their psychometric foundation (2, 11). One reason might be that such studies require greater time and personnel investments, which frequently result in smaller sample sizes, usually below *N* = 100 (2). According to older rules of thumb, sample sizes for factor analyses were generally recommended to exceed the number of variables multiplied by ten [12 cited in 13]; yet, these guidelines were not derived by theoretical or empirical knowledge and may prevent researchers from creating favorable statistical models. Meanwhile, several simulation studies (13, 14) have suggested that a minimum sample size of *N* = 60 is sufficient for a one-factor model assessed by seven items, like the single EAS scales, with low communalities (i.e., explained item variance by the factor ranging between .2 and .4). Similarly, testing the single scales of the structural model with the six scales used to measure an overall emotional availability score allows for an even smaller sample size. A greater sample size is need if one aims to test both the structural model along with all the single scales and accompanied items. However, simulation studies suggest that a multidimensional analyses for six independent factors measured by seven items each with high communalities (ranging between .6 and .8) require a sample size of only *N* = 110. Finally, because a smaller sample size would lower the power for rejecting a measurement or structural model, using a larger sample size is therefore a progressive approach to hypothesis testing, a larger sample size makes it easier to detect model violations.

Other psychometric characteristics like interrater reliability do not seem to be a core issue of the EAS (2, 15, 16). High internal consistency among the dimensions has also been repeatedly attested (17). However, high interrater reliability and high internal consistency are in agreement with a one-factor model, or, conversely, are results of a one-dimensional instrument.

### Research questions

Our intention of this psychometric analysis was to investigate the assumed underlying factor structure of the EAS based on emotional availability theory (8). We assumed that a child's age is a very important aspect in this matter, as parental behavior may depend on the child's level of development. For example, acting out structuring may be less important in a sample of infants and more important in a sample of preschool children. The results presented by Aran (7) may therefore be limited to infant samples. We therefore examined the factor structure of the parent and child scales, including the hypothesis of scale homogeneity on each of the six scales. This is preceded by an examination of item- and scale-based descriptive statistics and interrater reliabilities at item and scale level. If the factor structure is not supported by the data, we ask which structure is supported by an exploratory factor analysis. All planned tests are described in the Methods section below.

**Figure 1:**
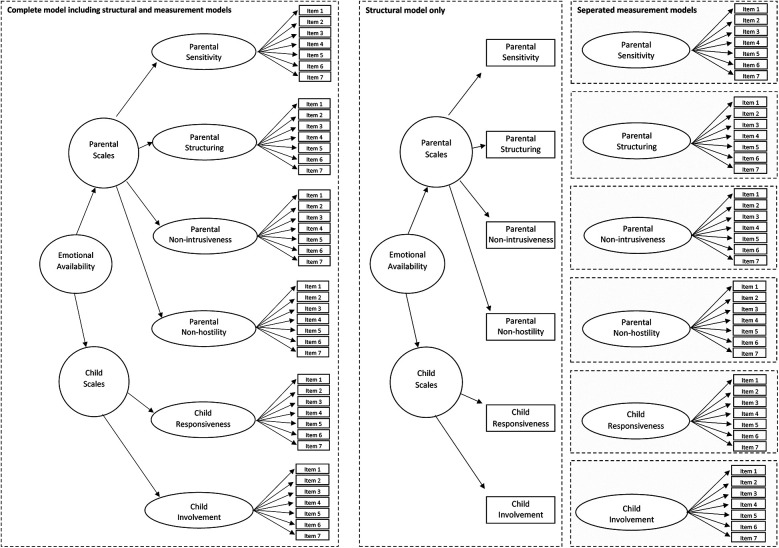
Left side: Complete model including structural and measurement models of the emotional availability scales. Mid: Structural model only without measurement models. Right side: Separated measurement model of all single scales.

A summary of the structural and psychometric hypothesis is given in Figure 1. First, we tested the hypothesis about model fit of the overall model presented in Figure 1 (left side) and, therefore, included the structural hypothesis as well as the six measurement models of the EAS in one global testing approach. Because we expected model violation, we examined a number of submodels and started our second hypothesis with a proof of the structural hypothesis in Figure 1 (mid), which was based solely on the scale scores, and we assumed the measurement models were psychometrically sound. The measurement models in Figure 1 (mid), were tested subsequently to investigate unidimensionality for each scale.

## Method

### Recruitment

The study was approved by the Ethics Committee of the Medical Association of the Physicians Chamber Westfalen-Lippe (AZ: 2013-620-f). Informed Consent was obtained for all participants in this study. Approximately 80% of the contacted parents agreed to study participation. The EAS scales 4th edition is generally applicable without exclusion criteria related to mental health problems in the version for preschool-aged children (1). However sufficient skills in the German language were necessary. Note that our sample consisted of a clinical and a non-clinical sample within a cross-sectional study design. The clinical sample was recruited during their treatment in the Family Day Hospital for preschool children, which is part of the Clinic for Child and Adolescent Psychiatry, Psychosomatics and Psychotherapy of the University Hospital Muenster between December 17, 2015 and October 5, 2016. To be treated in the Family Day Hospital, a child has to have at least one diagnosis of a behavioral or emotional disorder according to the International Statistical Classification of Diseases and Related Health Problems 10th Revision (ICD-10), while exclusion from treatment in the Family Day Hospital occurs when a child has a pervasive developmental disorder (F84; World Health Organization, 1992) or a parent has an acute mental illness requiring inpatient treatment. A description of the clinical treatment approach and the intake population sample of the Family Day Hospital has been described previously (18, 19).

The non-clinical sample was approached in the Family Day Hospital (20) in January 18, 2016 and March 31, 2018. Mothers of the non-clinical group were recruited from several day care centers in Münster. After receiving the study information and giving informed consent, the mother completed several questionnaires, including the Child Behaviour Checklist [CBCL (21)]. Eligibility required a score below the clinical cut-off on the CBCL, and two dyads were excluded. The remaining mother and child attended the Family Day Hospital, and participating dyads were videotaped interacting in a playroom with age-appropriate toys, e.g., a wooden train set (tracks, trains, barriers), knights, animals, small dolls to play with together.

### Sample

Overall, data from *N* = 116 parent-child dyads were analyzed in the current study, with the clinical sample accounting for *n* = 86. Of participating adults, 115 (99%) were female; parents' ages ranged between 18 and 56 years. The mean age of participating children was *M* = 4.61 years (SD = 1.61; 61.21% boys). It was a Western, educated, industrialized, rich, and democratic (WEIRD) sample. The most common types of main diagnoses at the time of admission were emotional disorders with onset specific to childhood (F93; 39%), other behavioral and emotional disorders with onset usually occurring in childhood and adolescence (F98; 24%), and conduct disorders (F91; 19%).

### Rating procedure

The participating dyads were recorded as they interacted in a free-play setting in a playroom, where age-appropriate toys, e.g., a wooden train set, were presented. To ensure observation of a natural and valid free-play interaction, dyads were instructed to play and enjoy their time together. Ten minutes of video material were then rated with regard to the observed emotional availability by two independent and, with respect to the clinical status of the children, blind raters. Raters completed a three-day online training as a requirement for application. The fee-based training costs $603 per person, and the obtained certificate is valid for two years. Both raters were certificated in terms of reliable application. The videos were rated in an individually randomized order by each rater between October 9, 2020 and August 18, 2022.

### EAS instrument and its scoring

The EAS instrument is not based on counting specific behaviors but on a global judgement additionally informed by the observers' clinical opinion, according to the guidelines in the manual (8). Four parental scales aim to assess the following aspects: sensitivity (authentic warmth, responsiveness to the child's cues) structuring (providing security, support, and guidance), non-intrusiveness (following the child's lead, absence of invasive actions), and non-hostility (absence of overt and covert negativity). Two child-related scales aim to assess responsiveness (authentic positive affect, responsiveness to the adult) and involvement (positive initiation of interaction, balanced autonomy and inclusion). Each of the six scales is represented by seven items: The first two items are scored on a seven-point rating scale, while five complementary items are scored on a three-point rating scale. Therefore, the score of each scale, as the sum score of all seven items in a scale, ranges from seven to 29.

### Planned analysis

We calculated the basic descriptives of mean and standard deviation based on the original ratings. On this basis, interrater reliability on the item level was computed to examine the statistical precondition of any bi- and multivariate analysis. To weight each rater equally, the ratings were standardized prior to computing aggregated scores for all subsequent analyses. This standardization excludes the measurement error of rater differences in order to avoid the possibility that the model test fails not because of a deviation in the measurement structure, but because of rater influences, which could confound the test of structural validity. Note that the investigation of rater influences—which is beyond the scope of this analysis—is rather complex and can best be investigated after factorial validity has been confirmed. Nevertheless, the results should be interpreted bearing in mind that we have excluded rater differences that become relevant in clinical practice. The basic description of aggregated item means and standard deviations was repeated on the scale level consulting the aggregated scores. We additionally reported Cronbach's alpha.

We have not excluded any data and refer to the simulation studies mentioned in the introduction to ensure a sufficient minimum sample size. A preliminary analysis with a smaller sample was performed by the second author on February 27, 2021, and the results of this analysis along with the model specification in (7) led to a detailed conceptualization of the EAS structure and related model testing. Notably, our analysis includes model testing on the item level, which gives additional insight into the measurement model for each scale. The EAS coding material is copyright protected and is only assessable via Zeynep Biringen. Note that because of possible violation of copyright, Aran (7) did not report detailed results on the item level.

### Test of structural validity and unidimensionality of the single scales

To examine the model's structural validity, we first tested the complete model (Figure 1, left side), after which we tested the path model (Figure 1, mid) of the structural model, assuming that the six measurement models (Figure 1, right side) are psychometrically sound. Subsequently, each of the six measurement models (Figure 1, right side) was tested by a confirmatory factor analysis to locate potential sources of model violations. The model testing was conducted with SAS PROC CALIS (SAS 9.4; Statistical Analysis Systems; CALIS = covariance analysis of linear structural equations) to evaluate model fit according to Schermelleh-Engel (22). The parameter estimation was conducted by maximum likelihood (ML) but repeated with SAS robust and OLS (ordinary least squares) estimation. Because the results from these two approaches essentially did not differ, only the results from the ML method are presented.

### Explorative factor analysis for *post hoc* analysis

After model testing, we aimed to explore the cause of psychometric inconsistencies identified earlier. We, therefore, carried out a *post hoc* analysis to extract the number of factors determined by the 42 EAS items. We compared the observed eigenvalue of each factor with eigenvalues estimated based on random numbers by applying the SAS program for determining the number of components using parallel analysis by Velicer's minimum average partial test, which focuses on the common variance in a correlation matrix test, as done by O'Connor (23). In a second step, we described the pattern of item loadings according to the factors determined in step one, and we evaluated whether the new factors could be interpreted according to emotional availability theory (8).

Additionally, and with knowledge of ceiling effects, we conducted a further literature search to examine whether ceiling effects were unique to our data or were also reported in other publications. Finally, we computed the descriptive test score PDTS (probability of distinct test scores) (24, 25), which reports the average probability of distinguishing between two randomly selected test scores using the critical differences for each EAS dimension separately, as this probability can be lowered when ceiling effects are observed. In these cases, the PDTS constitutes a superior parameter compared to Cronbach's alpha, which can be high even if the test score distribution shows ceiling effects. As ceiling effects, in general, mean that a number of test scores in a sample are located in a small range, the PDTS performs a complete comparison on any two test scores in a sample and returns the number of significant comparisons divided by the number of all comparisons. Therefore, the PDTS score can vary between 0% and 100%, and it is lower when the test score distribution shows ceiling effects. We interpreted the PDTS index according to (26) as “very poor” < 30%; “poor” = 30%–45%; “moderate” = 45%–60%; “good” = 60%–75%; “very good” = 75%–90%, and “excellent” > 90%.

## Results

### Item and scale descriptives

Table 1 shows the rating format, means, and standard deviations on the item level for rater A and rater B based on the original unstandardized scale scores and the Pearson correlation between rater A and B along with the part-whole corrected item-to-scale correlation. In Table 2 we present means and standard deviations of the original unstandardized scale scores on the scale level along with means and standard deviations for the clinical and non-clinical sample. These values reveal ceiling effects on the item level, especially for items with the three-point rating format, as well as on the sum score level. Table 2 additionally includes Cronbach's alpha, the PDTS score, and the correlation between the scale scores. Note that the reported correlations between scale scores are not attenuated by the scales' reliability, which may lead to an underestimation of these correlations. The attenuation is an inherent adjustment within an SEM approach.

**Table 1:** Emotional availability scales (EAS) item descriptives for 42 items, showing the range of answer formats, means, standard deviations, part-whole corrected item-to-scale correlation based on aggregated ratings from rater A and rater B, and correlation between raters A and B.

Observed dyad members	Scale	Item	*Min–Max*	Mean	SD	*r*_it_	*r*_AB_
Parent/Caregiver	Sensitivity	1	*1–7*	5.39	1.14	0.89	0.58
		2	*1–7*	5.26	1.18	0.89	0.54
		3	*1–3*	2.61	0.53	0.76	0.42
		4	*1–3*	2.50	0.51	0.85	0.44
		5	*1–3*	2.85	0.33	0.70	−0.17
		6	*1–3*	2.83	0.33	0.48	0.67
		7	*1–3*	2.83	0.39	0.62	0.62
	Structuring	1	*1–7*	5.37	1.16	0.91	0.42
		2	*1–7*	5.42	1.15	0.81	0.56
		3	*1–3*	2.61	0.48	0.76	0.53
		4	*1–3*	2.76	0.38	0.65	0.61
		5	*1–3*	2.92	0.22	0.44	0.74
		6	*1–3*	2.66	0.48	0.76	0.41
		7	*1–3*	2.78	0.41	0.66	0.03
	Intrusiveness	1	*1–7*	5.64	1.26	0.89	0.57
		2	*1–7*	5.94	1.13	0.91	0.48
		3	*1–3*	2.72	0.42	0.78	0.15
		4	*1–3*	2.68	0.39	0.78	0.22
		5	*1–3*	2.53	0.52	0.68	0.44
		6	*1–3*	2.70	0.44	0.83	0.15
		7	*1–3*	2.80	0.34	0.68	−0.07
	Hostility	1	*1–7*	5.62	1.21	0.76	0.52
		2	*1–7*	6.32	0.87	0.69	0.23
		3	*1–3*	2.98	0.15	0.34	1.00
		4	*1–3*	2.64	0.44	0.65	0.04
		5	*1–3*	2.87	0.33	0.68	−0.26
		6	*1–3*	2.78	0.37	0.35	0.16
		7	*1–3*	2.95	0.21	−0.05	0.89
Child	Responsiveness	1	*1–7*	5.35	1.09	0.87	0.60
		2	*1–7*	5.34	1.22	0.87	0.69
		3	*1–3*	2.84	0.29	0.45	−0.10
		4	*1–3*	2.71	0.49	0.79	0.54
		5	*1–3*	2.89	0.19	0.41	0.13
		6	*1–3*	2.67	0.44	0.87	0.27
		7	*1–3*	2.60	0.45	0.68	0.35
	Involvement	1	*1–7*	5.64	1.15	0.93	0.56
		2	*1–7*	5.21	1.32	0.92	0.66
		3	*1–3*	2.64	0.46	0.86	0.28
		4	*1–3*	2.84	0.31	0.21	−0.10
		5	*1–3*	2.69	0.41	0.84	0.16
		6	*1–3*	2.73	0.46	0.83	0.64
		7	*1–3*	2.76	0.38	0.69	0.52

**Table 2:** Emotional availability scales (EAS) scale descriptives with range of scales from a minimum of 7 to a maximum of 29, sum scale scores for raters A and B, mean differences between A and B by *t*-test, correlation between sum scale scores for raters A and B, and correlation between the aggregate sum scale scores of raters A and B for the complete, clinical and community sample of *N* = 116.

Sample	Scale	Rater A		Rater B		Mean Diff A–B	Corr	Aggregate A + B		Alpha	PDTS in %	Scale-to-scale correlation					
		*M*	SD	*M*	SD	p(t)^b^	Rater A, B	*M*	SD			(1)	(2)	(3)	(4)	(5)	(6)
Complete sample	(1) Sensitivity	23.91	4.53	24.58	3.67	.031	.69	24.25	3.77	.91	51.86		.85	.85	.87	.80	.76
	(2) Structuring	24.22	4.79	24.78	3.32	.114	.62	24.50	3.66	.90	49.04			.64	.70	.72	.67
	(3) Intrusiveness	24.61	4.50	25.44	4.15	.010	.69	25.02	3.98	.94	54.54				.72	.70	.69
	(4) Hostility	26.35	3.15	25.97	2.83	.080	.71	26.16	2.76	.77	23.76					.58	.52
	(5) Responsiveness	24.87	4.30	23.96	3.49	.002	.69	24.41	3.58	.89	46.99						.96
	(6) Involvement	24.97	4.79	24.09	3.77	.008	.69	24.53	3.95	.92	51.51						
Clinical sample	(1) Sensitivity	23.06	4.69	24.12	3.91	.008	.67	23.58	3.92	.85	40.96		.83	.84	.86	.76	.72
	(2) Structuring	23.65	5.04	24.38	3.37	.095	.59	24.02	3.78	.88	43.21			.60	.69	.67	.61
	(3) Intrusiveness	23.70	4.74	24.88	4.52	.005	.59	24.29	4.23	.86	39.49				.70	.66	.65
	(4) Hostility	25.82	3.42	25.68	3.11	.613	.60	25.75	3.01	.73	22.20					.54	.47
	(5) Responsiveness	24.16	4.50	23.53	3.42	.098	.68	23.85	3.60	.86	43.52						.95
	(6) Involvement	24.26	5.11	23.66	3.76	.151	.71	23.96	4.06	.92	51.51						
Non- clinical sample	(1) Sensitivity	26.35	2.93	25.92	2.47	.031	.48	26.13	2.54	.82	33.10		.87	.81	.89	.87	.86
	(2) Structuring	25.85	3.58	25.92	2.96	.114	.52	25.88	2.92	.85	38.62			.79	.77	.85	.86
	(3) Intrusiveness	27.22	2.20	27.03	2.18	.010	.49	27.13	2.04	.83	32.41				.68	.86	.85
	(4) Hostility	27.87	1.37	26.80	1.56	.080	.36	27.33	1.32	^a^	^a^					.73	.73
	(5) Responsiveness	26.92	2.83	25.17	3.49	.002	.58	26.04	3.03	.83	28.51						.98
	(6) Involvement	27.03	2.96	25.33	3.59	.008	.68	26.18	3.10	.85	31.49						

Mean Diff A–B, *t*-test repeated measurement; Corr, pearson correlation; Alpha, Cronbach's alpha; PDTS, descriptive test score that gives the average probability of distinguishing between two randomly selected test scores by the critical difference (24, 25).

^a^ Not computable.

^b^ Two-sided.

### Model testing

In Table 3 we report model fit indices based on maximum likelihood estimates for the structural model as well as all tested measurement models depicted in Figure 1. Note that we replicated all statistical tests with robust ordinary least square parameter estimates in order to address the non-normally distributed data, but we did not observe differing results. Moreover, the estimation of path coefficients in the structural model exceeded, in general, a level of .80, indicating a solution with high communalities. The path coefficients in the measurement models varied considerably. For the sensitivity model, the path coefficients ranged from .54 to .96, for structuring from .37 to 1.00, for non-intrusiveness from .71 to .96, for non-hostility from −.06 to .89, for responsiveness from .42 to .92, and for involvement from .26 to .96; most path coefficients fell in the range of .75–.95. This, in turn, indicates, as mentioned in the simulation studies (13, 14), that our study had a sufficient number of observations for the intended model testing. Overall, all conducted tests revealed misfits and, therefore, suggest rejection of the models.

**Table 3:** Model fit indices of emotional availability models according Figure 1, including the complete, the structural and the six measurement models with *N* = 116 estimated by SAS PROC CALIS.

Model	Items	Chi-square	df	Pr > Chi-square	RMSEA	Model acceptance
Complete model	42	5,829.67	793	<.0001	0.235	Rejected
Structural model	6	213.679	9	<.0001	0.445	Rejected
Adult Sensitivity	7	55.399	14	<.0001	0.160	Rejected
Adult Structuring	7	43.060	14	<.0001	0.134	Rejected
Adult Intrusiveness	7	34.863	14	<.0001	0.113	Rejected
Adult Hostility	7	25.589	14	0.0292	0.084	Rejected
Child Responsiveness	7	35.725	14	0.0011	0.116	Rejected
Child Involvement	7	47.976	14	<.0001	0.145	Rejected

According to (22), the model fit is acceptable if RMSEA ≤ .08.

### *post hoc* exploratory factor analysis

**Figure 2:**
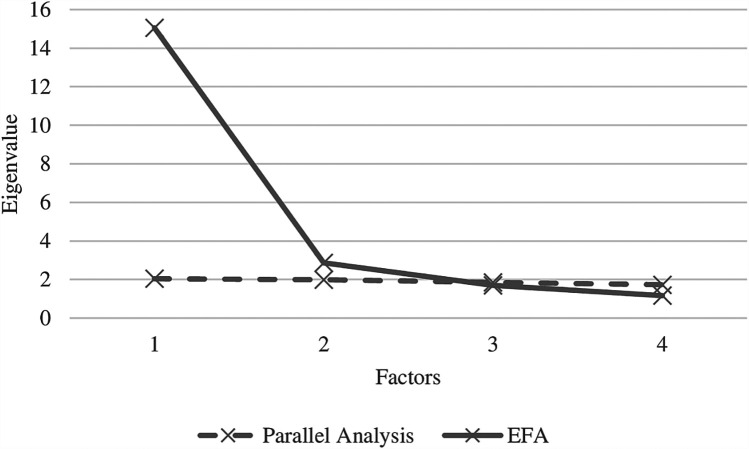
Exploratory factor analysis (EFA) scree plot on all 42 items of the emotional availability scales (solid line) in comparison with the scree plot of 42 items with random numbers according Velicer's minimum average partial (MAP), which is the upper confidence interval (95th percentile) of randomly generated eigenvalues for the same sample size.

In order to evaluate *post hoc* whether the number of factors in the EAS model was mainly responsible for the model rejections, we conducted a parallel analysis that estimated eigenvalues based on random answers. Based on simulation studies, we computed Velicer's minimum average partial (MAP), which is the upper confidence interval (95th percentile) of randomly generated eigenvalues for a given sample size in combination with the number of items in a real data set, with SAS-Macro software (23). The comparison of eigenvalues based on random vs. real data is displayed in Figure 2, and it suggests that the EAS item covariation can be best described by two factors. To get an impression of the item loading pattern in a two-factorial solution, oblique (not orthogonal) rotation was used, which allows the factors to be correlated. This is justified if the first factor is considered to represent parental characteristics while the second factor contains the child characteristics, with both factors being expected to be correlated. However, the new first dimension consisted of 18 parental and two child items, while the new second dimension consisted of 12 parental and 12 child items (see Table 4). Given the theoretical superordinate distinction of parental and child items by Biringen, we were not able to interpret this pattern.

**Table 4:** *post hoc* item loadings from an exploratory factor analysis with *N* = 2 factors.

Observed dyad members	Scale	Item	1. Factor	2. Factor
Adult	Sensitivity	1	.72	.29
		2	.50	.55
		3	.43	.56
		4	.55	.43
		5	.67	.06
		6	.30	.48
		7	.65	.06
	Structuring	1	.67	.24
		2	.64	.36
		3	.64	.08
		4	.63	.16
		5	.43	.18
		6	.68	.14
		7	.45	−.06
	Intrusiveness	1	.29	.55
		2	.31	.55
		3	.38	.34
		4	.36	.47
		5	.68	.23
		6	.31	.39
		7	.16	.50
	Responsiveness	1	.74	.11
		2	.71	.01
		3	.35	−.04
		4	.66	.00
		5	.69	.04
		6	.31	.39
		7	.03	.01
Child	Involvement	1	.19	.79
		2	.16	.83
		3	.07	.45
		4	−.02	.92
		5	.37	.02
		6	−.03	.89
		7	−.11	.81
	Hostility	1	.03	.93
		2	.05	.92
		3	.03	.86
		4	.50	−.01
		5	−.08	.90
		6	−.01	.90
		7	−.26	.96

Higher item loadings on one factor are marked.

## Discussion

This study examined the psychometric foundation of the 4th edition EAS (1). Our findings for a sample of preschool-aged children and their mothers were not in agreement with the underlying theoretical expectations. In its current version, none of the six scales—neither the superordinate parental, child-related, nor a global emotional availability factor—showed an acceptable model fit. Our interpretation that EAS scores lack a psychometric foundation is in agreement with Aran (7) and their results reported for a sample of infants. The main reason for the rejection of the model is revealed by the *post hoc* analysis using exploratory factor analysis, which identified only two factors instead of six. In addition, these two factors each included a mixture of both parental and child-related items. Thus, as the six scales of the EAS contain items that were assigned to two different factors, this led to the rejection of one-dimensional models. Note that the majorities of our observed commonalities were mainly between 0.6 and 0.8, which is considered a “wide” commonality condition [see Mundfrom (13)], and with 7 indicators per factor this requires a minimum sample size of *N* = 110 for 6 factors to be confirmed at an excellent level criterion (0.98)—which is the case for the EAS, and for a good level criterion (0.92) only *N* = 55 observations are required.

Our findings additionally explain the high correlations among the scales, which have previously been observed in many studies applying the EAS (27). The results should not be overinterpreted and do not generally call into question the terms related to parental sensitivity, structuring, intrusiveness, or hostility or to child responsiveness and involvement, nor do they question their testability or distinction. The shortcomings of the EAS are more likely to be attributed to the limitations of test development, in particular the generation of a large item pool, a repeated item selection process guided by factor and structural models to locate items on the intended content or, finally, scales of facets of emotional availability. The review by Lotzin (2) provides an overview of a number of alternative observation tools. However, Lotzin mentioned that the majority of observational measures have also not been developed with the help of psychometric methods, probably because old rules of thumb suggest large sample sizes before conducting a factor analysis; such sample sizes can hardly be achieved for observational studies given the effort needed to recruit clinical samples, which requires an enormous investment in personal and room facilities plus double-rated, blinded, and trained observers. A positive exception is the work of Wilson and Durbin (28), which was based on results of a factor analysis, did not require training, and assessed five parenting scales (involvement, positivity, hostility, intrusiveness, discipline). However, given the empirical and conceptual overlap with previous terms introduced from Baumrind (29), like “parental control,” as well as newer developments [see Grolnick, (30)] and additional constructs mentioned in Pritchett (10), experts in the field are still faced with a multitude of terms and construct and misses an integrated and consistent thesaurus. Therefore, many more studies on observational instruments in the field are needed to provide evidence regarding their internal structure (Standard 1.13, p. 26), their relationships with conceptually related constructs (Standard 1.16), and their relationships with criteria [Standard 1.17; all Standards defined by the American Educational Research Association, American Psychological Association, & National Council on Measurement in Education, (31)]. Once a researcher can use a structurally valid observation tool, many subsequent issues related to the application can be investigated, such as participant characteristics, settings, instructions, and the assessment procedure, including live or video-based observation, duration of observation, or training (32).

### Findings beyond structural validity: ceiling effects, interrater reliability and application in practice

Our mean scale scores appeared around the upper quarter of the scale range, which is not a specific effect of the non-clinical participant. Such ceiling effects have been detected in other studies across different conditions, e.g., for shorter [5 min (33)] and longer observation times [25 min (7, 15)], across stressful and non-stressful situations (33), and with infants (7, 34) as well as toddlers (16, 35) and preschool-aged children (36). The identified effects are, therefore, not unique to our sample, setting, or duration time, and such effects also depend on the changing rating format across and within different versions of the EAS. Note that there is a certain robustness for test statistics to non-normality like ceiling effects in confirmatory factor analysis (37, 38), and all model tests were repeated with robust estimates in SAS PROC CALIS, which did not lead to differing results. In general, the EAS appears to be more appropriate for clinical samples and therefore we do not expect the relatively small number of non-clinical participants to limit the main conclusions of the study.

Do the ceiling effects limits the applicability of the EAS? A first impression of the internal consistency (Cronbach's alpha) seems to be sufficient and in a comparable range as reported in other publications (17). However, Cronbach's alpha does not reflect limitations related to ceiling effects, which became more visible when computing the alternative test score PDTS. In our sample, the average probability of distinguishing between two test scores ranged from “very poor” (22.2% for non-hostility) to “moderate” (54.54% for non-intrusiveness), which leads to a practical limitation in using the EAS scores in a clinical context.

Interrater reliability is another issue that affects the applicability of an observational tool while our main intention was the examination of the structural validity. Therefore our analysis were based on the raters aggregate and exclude this source of measurement error. However a short note is given to our results before we aggregated. We observed mean score differences between both raters for scores on sensitivity, non-intrusiveness, and responsiveness, along with correlations ranging from *r* = .62 (structuring) up to *r* = .71 (non-hostility). Such rater influences have been reported previously (39). The analysis of rater differences is rather complex and was handled by building an aggregate score to exclude this source of error from our intended analysis, which focused on item and scale properties.

### Constraints on generality

The results presented in our study are limited by the clinical sample taken at admission from the Family Day Hospital for preschool-aged children in a child and adolescent psychiatry unit, and the non-clinical sample from a midsize city in Germany. Further, the recruiting procedure excluded severe developmental disorders. The observed scores were based on observation in a setting of free-play interaction for a limited duration of observation.

## Summary

The Emotional Availability Scales (EAS) is a multidimensional observational instrument that is used to understand the emotional connection between parents and young children. Our analysis found statistical problems with the psychometric foundation scale scores, indicating that the suggested six scales are not supported by the data, corresponding to findings by Aran et al. (7). We found that a two-factor model, not a six-factor model as represented by the six scales of the EAS, fit best to the data, but the two-factor model was difficult to interpret. However, some important concepts covered by the EAS still seem valuable in clinical practice and can potentially be assessed using alternative measures.

## Data Availability

The datasets presented in this study can be found in online repositories. The names of the repository/repositories and accession number(s) can be found below: Müller, J. M. (2024). EAS data from two raters for a sample of preschool aged children. https://doi.org/10.23668/psycharchives.14072; Müller, J. M. (2024). SAS EAS psychometric analysis, two raters, SEM model. https://doi.org/10.23668/psycharchives.14044.
