# Supplementary material for: Psychometric analysis of the emotional availability scales for video-recorded interactions between parents and their preschool-aged children
Source: Front Child Adolesc Psychiatry. 2025 Apr 9;4:1528196. doi: 10.3389/frcha.2025.1528196 (PMC12014617; doi:10.3389/frcha.2025.1528196)
Supplement: Supplementary file 1 [file Datasheet1.docx]

Supplementary Material:

Model and parameter estimation with ML, robust and OLS for a one-factor (left column) and two-factor (adult and child factor) model (right column)

| SEM with SAS PROC CALIS (SAS 9.4) similar to Aran et al. (2022) One factor - Estimation with ML, robust and OLS | SEM with SAS PROC CALIS (SAS 9.4) similar to Aran et al. (2022) - two factor (Adult + Child Factor) Estimation with ML, robust and OLS |
| --- | --- |
| 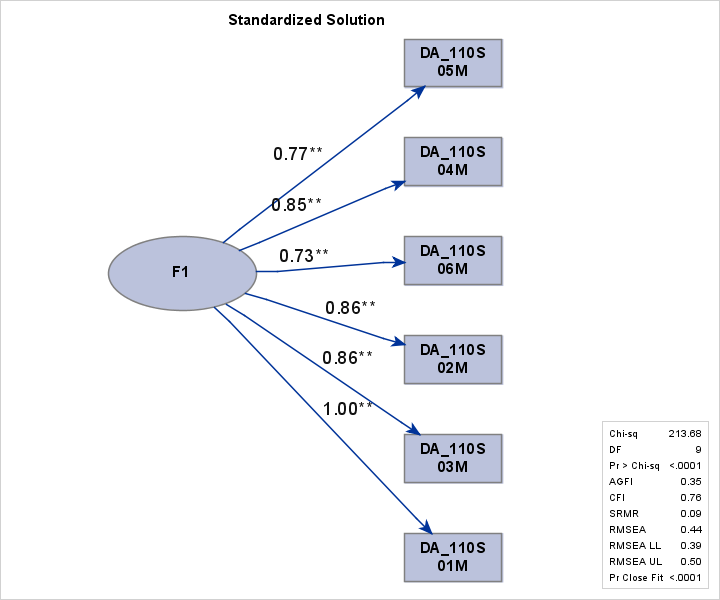 | 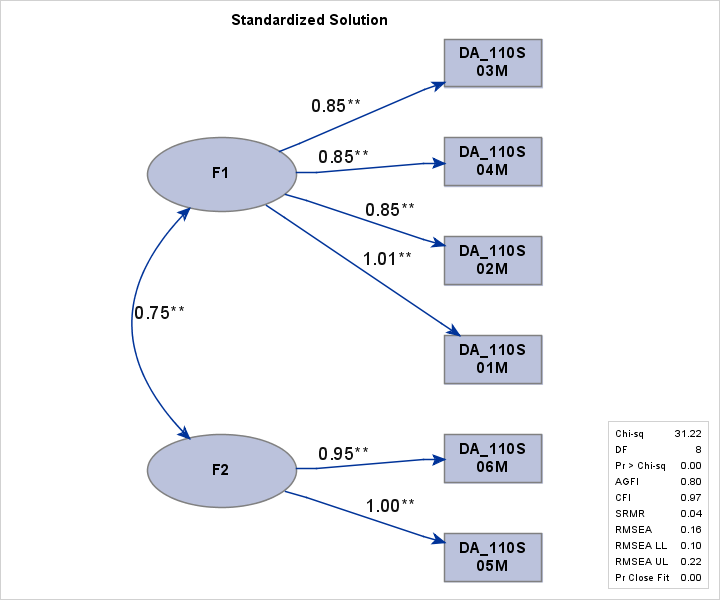 |
| 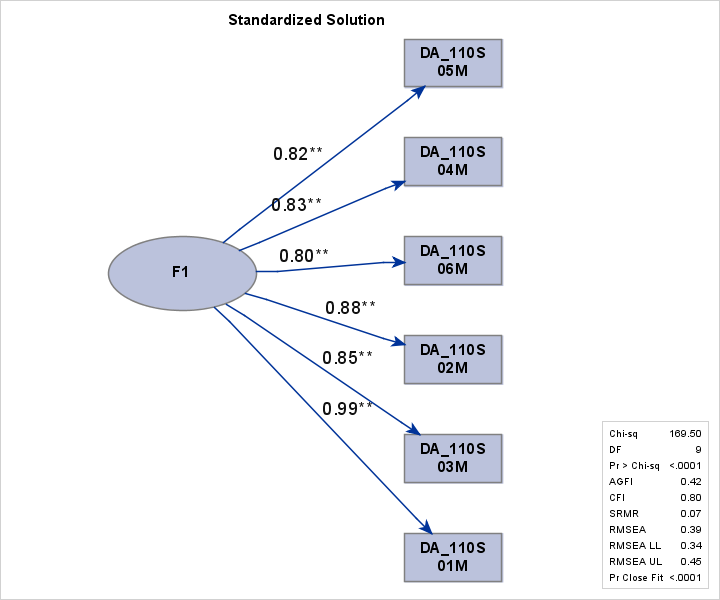 | 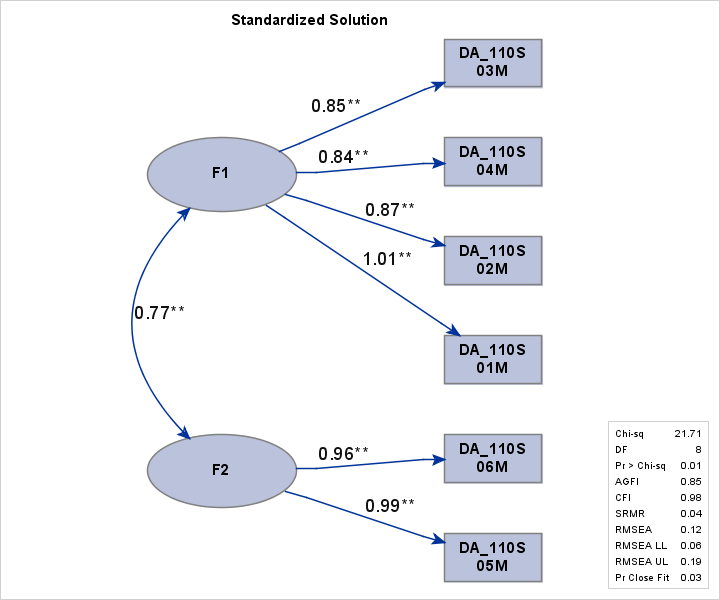 |
| 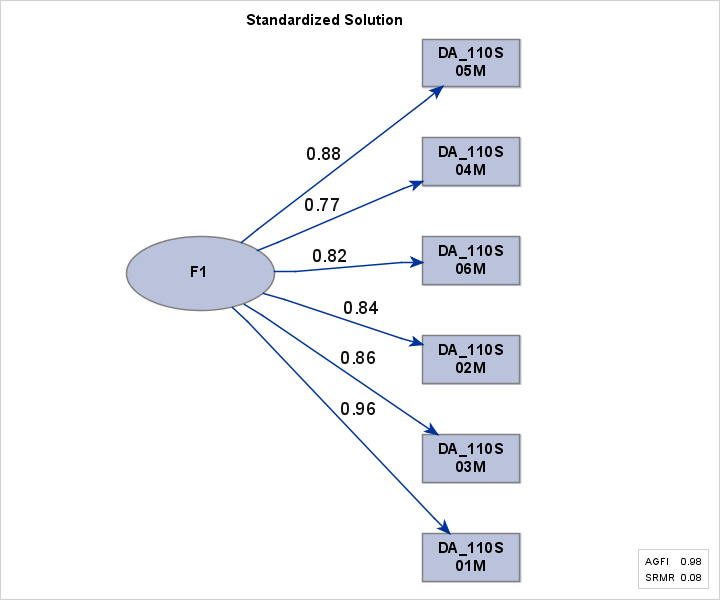 | 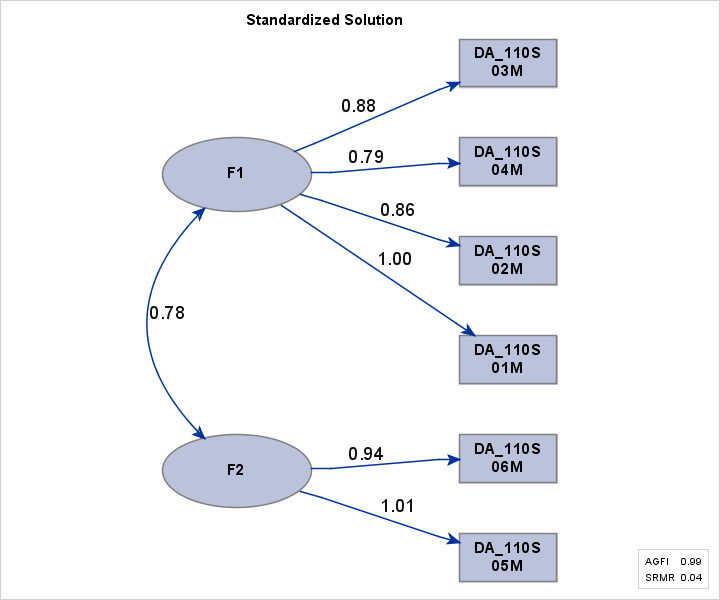 |

Confirmatory factor analysis for the six EAS scales (F1-F6) with ML estimation, and repeated estimation with SAS PROC CALIS (SAS 9.4) robust option and OLS estimation.

| ML estimation | |
| --- | --- |
| 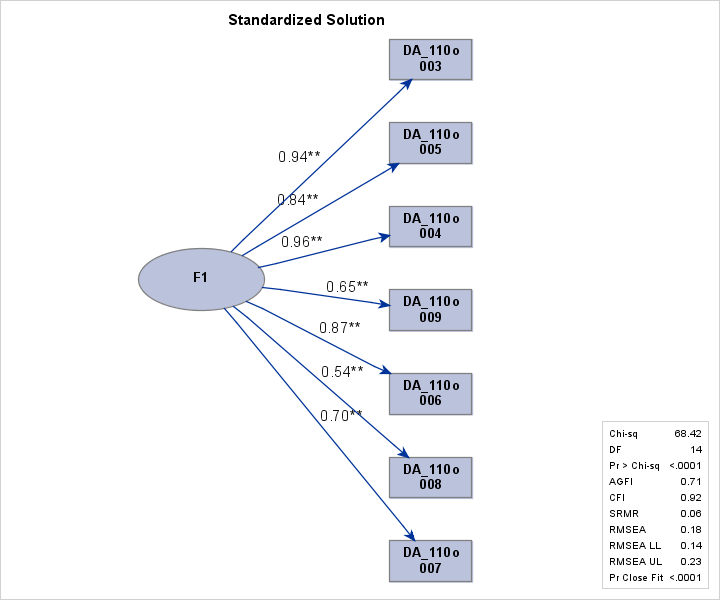 | 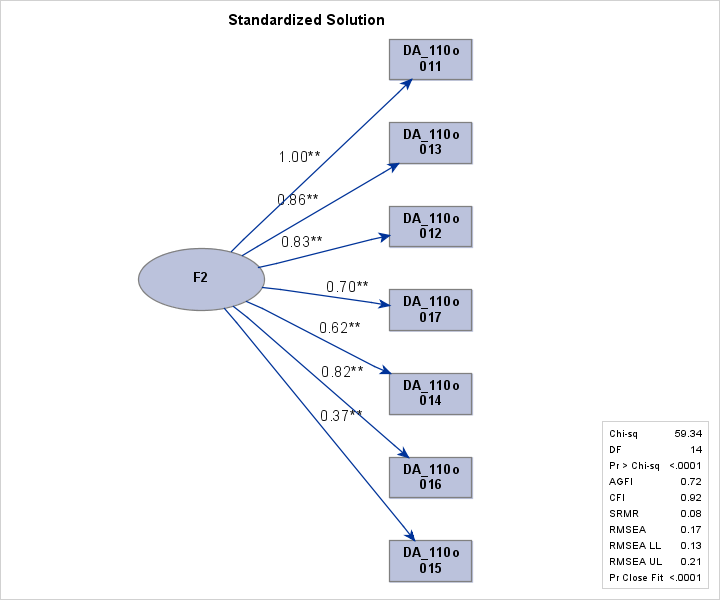 |
| 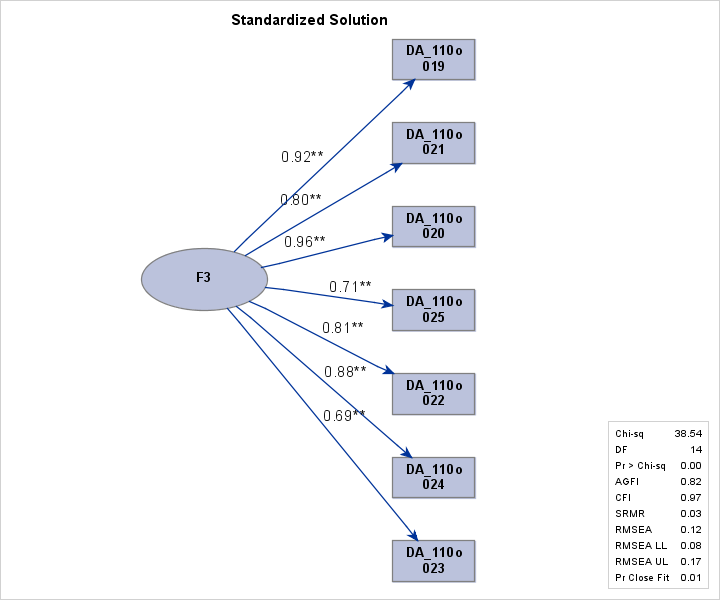 | 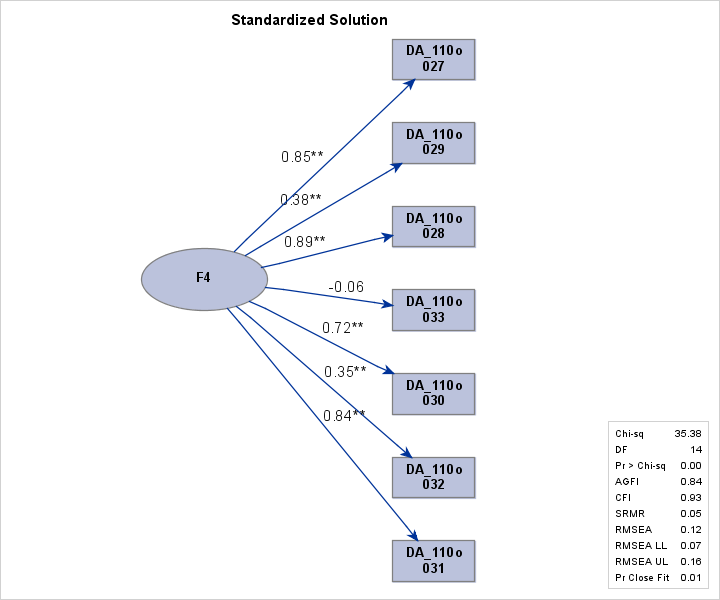 |
| 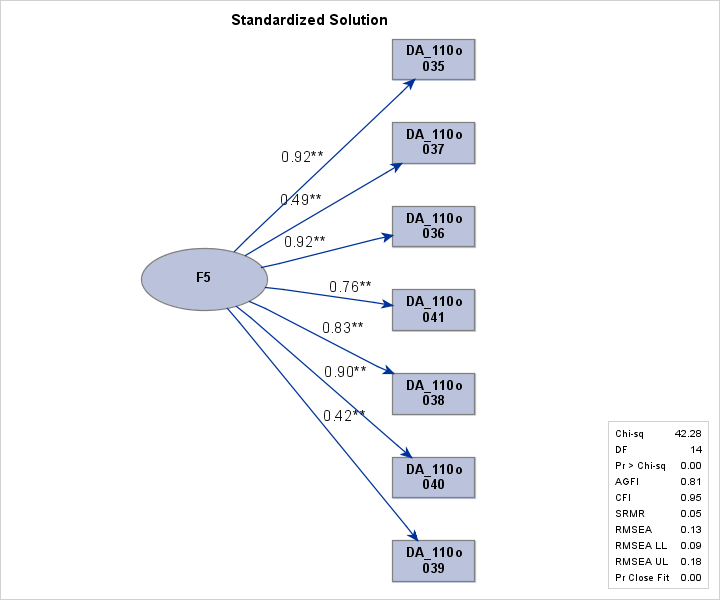 | 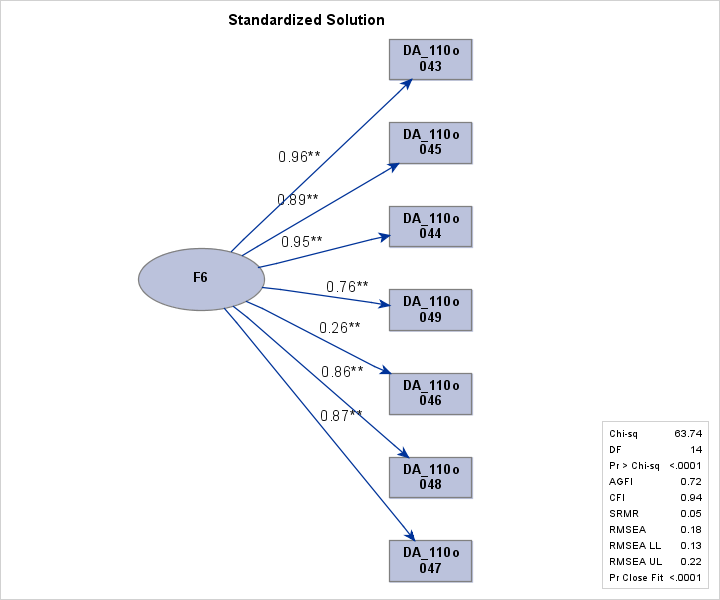 |

| Robust estimation option in SAS PROC CALIS (SAS 9.4) | |
| --- | --- |
| 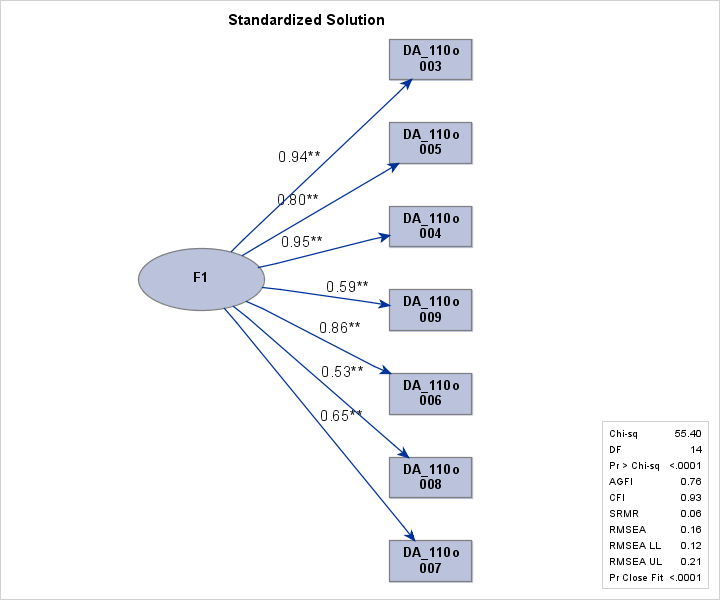 | 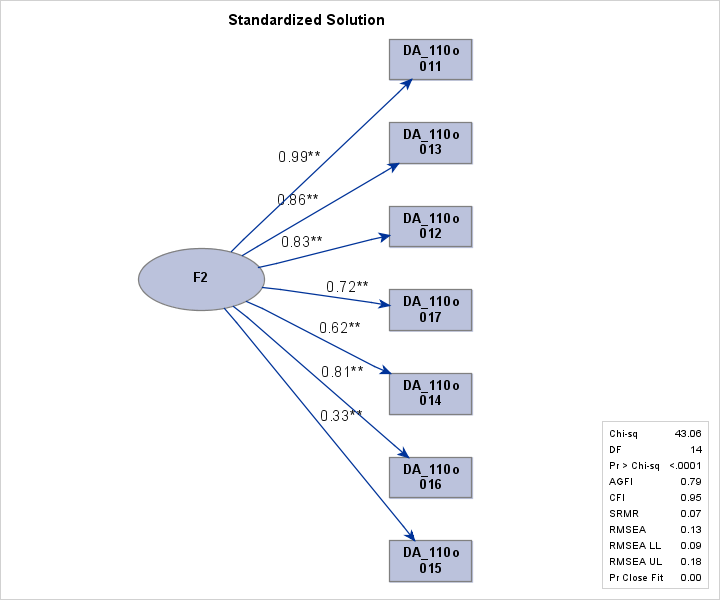 |
| 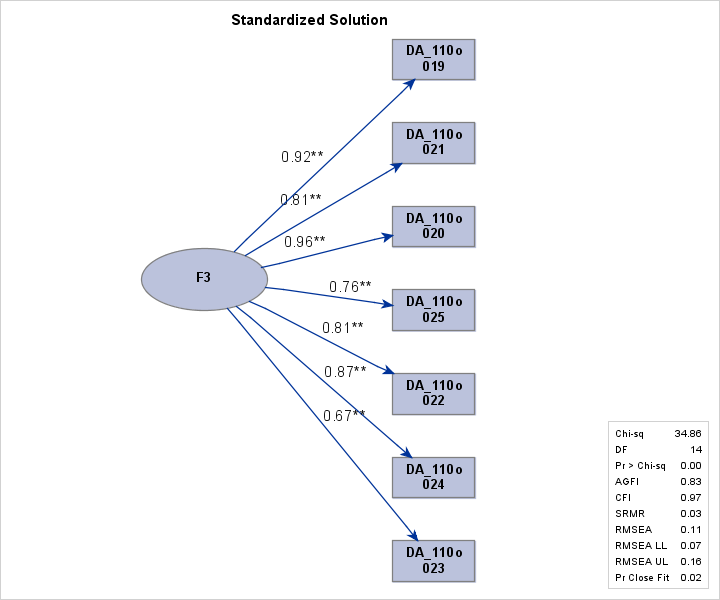 | 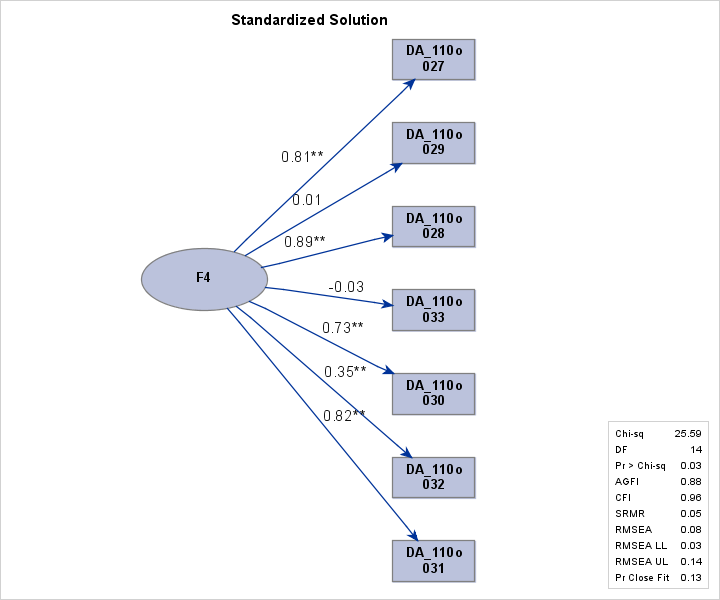 |
| 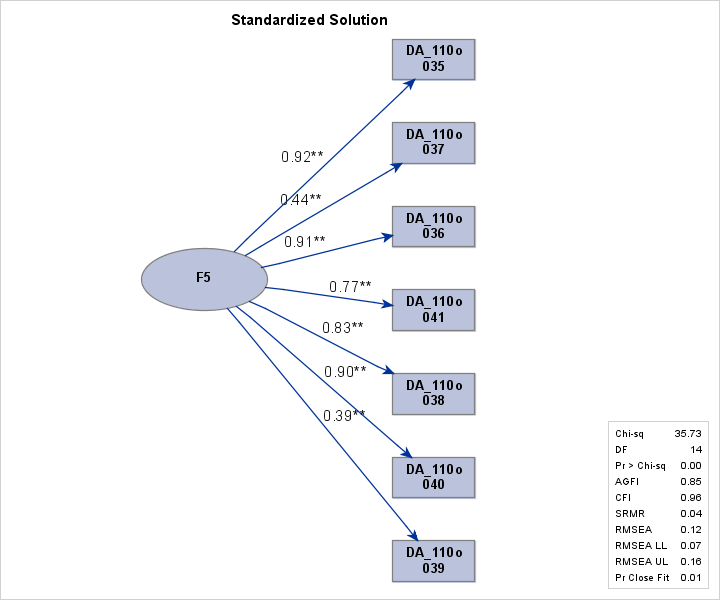 | 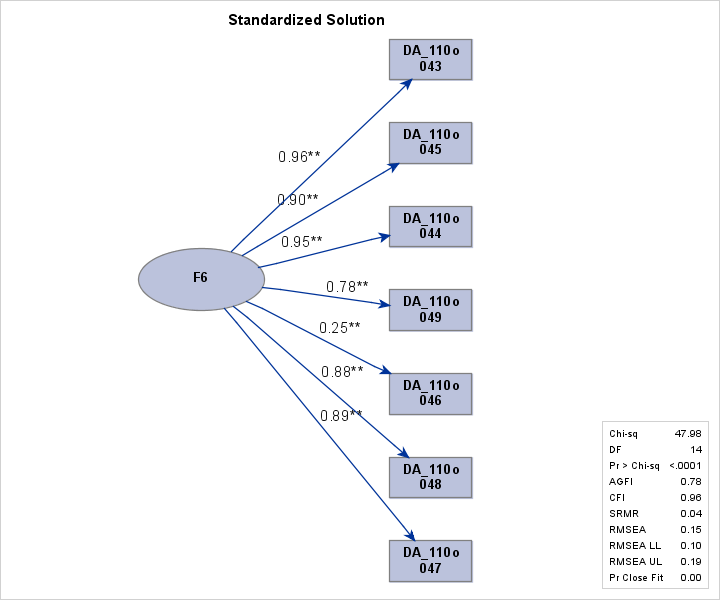 |

| Robust OLS estimation with SAS PROC CALIS (SAS 9.4) | |
| --- | --- |
| 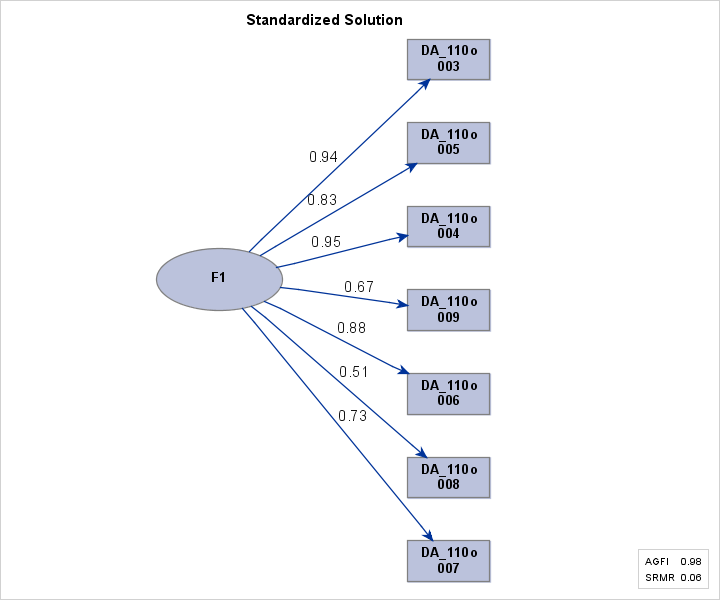 | 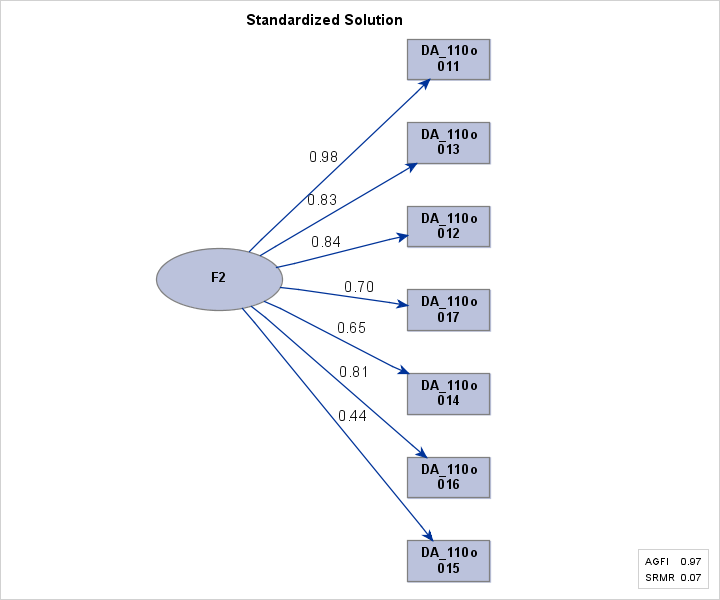 |
| 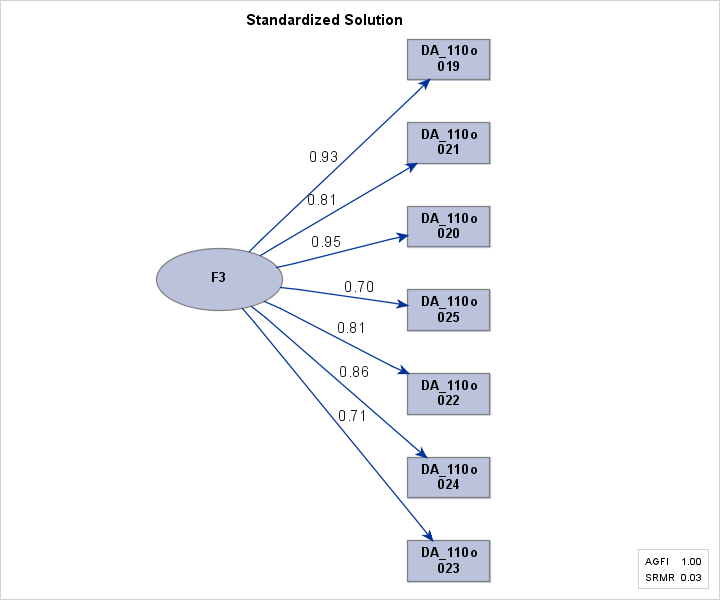 | 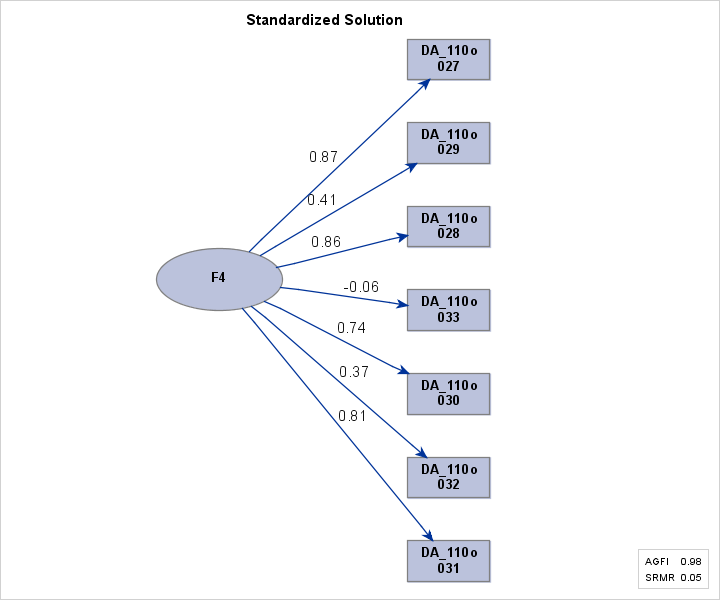 |
| 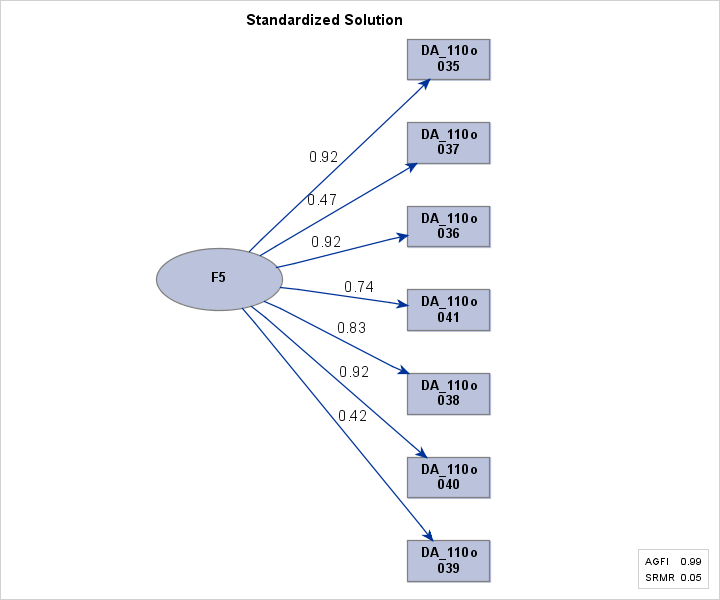 | 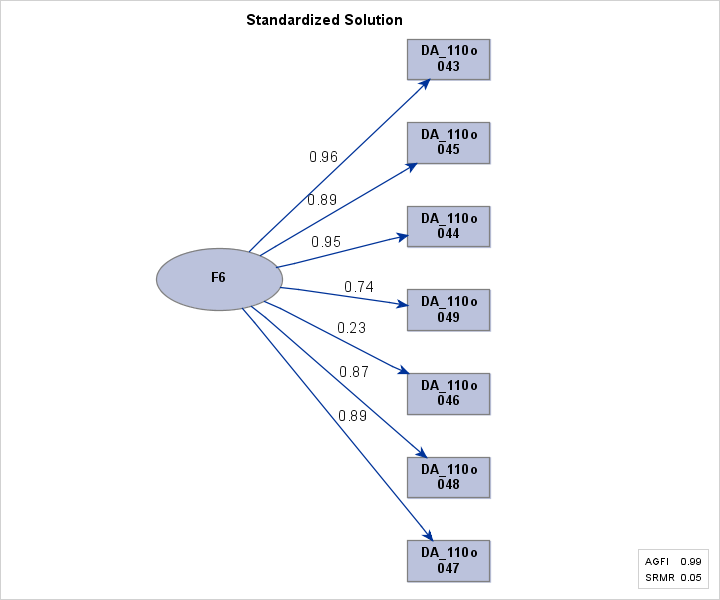 |
